# Analysis of M4 Transmembrane Segments in NMDA Receptor Function: A Negative Allosteric Modulatory Site at the GluN1 M4 is Determining the Efficiency of Neurosteroid Modulation

**DOI:** 10.3389/fphar.2021.769046

**Published:** 2021-10-01

**Authors:** Kai Langer, Adriana Müller-Längle, Jannik Wempe, Bodo Laube

**Affiliations:** ^1^ Department of Neurophysiology and Neurosensory Systems, Technische Universität Darmstadt, Darmstadt, Germany; ^2^ Centre for Synthetic Biology, Technical University of Darmstadt, Darmstadt, Germany

**Keywords:** N-methyl-D-aspartate receptor, excitatory glycine receptor, M4, transmembrane segment interactions, pregnenolone sulfate, negative allosteric modulator

## Abstract

Ionotropic glutamate receptors (iGluRs) are tetrameric ligand-gated ion channels that play a crucial role in excitatory synaptic transmission in the central nervous system. Each subunit contributes with three helical transmembrane segments (M1, M3, and M4) and a pore loop (M2) to form the channel pore. Recent studies suggest that the architecture of all eukaryotic iGluRs derives from a common prokaryotic ancestral receptor that lacks M4 and consists only of transmembrane segments M1 and M3. Although significant contribution has emerged in the last years, the role of this additionally evolved transmembrane segment in iGluR assembly and function remains unclear. Here, we have investigated how deletions and mutations of M4 in members of the NMDA receptor (NMDAR) subfamily, the conventional heteromeric GluN1/GluN2 and glycine-gated GluN1/GluN3 NMDARs, affect expression and function in *Xenopus* oocytes. We show that deletion of M4 in the GluN1, GluN2A, or GluN3A subunit, despite retained receptor assembly and cell surface expression, results in nonfunctional membrane receptors. Coexpression of the corresponding M4 as an isolated peptide in M4-deleted receptors rescued receptor function of GluN1/GluN2A NMDARs without altering the apparent affinity of glutamate or glycine. Electrophysiological analyses of agonist-induced receptor function and its modulation by the neurosteroid pregnenolone sulfate (PS) at mutations of the GluN1-M4/GluN2/3-transmembrane interfaces indicate a crucial role of position M813 in M4 of GluN1 for functional coupling to the core receptor and the negative modulatory effects of PS. Substitution of residues and insertion of interhelical disulfide bridges confirmed interhelical interactions of positions in M4 of GluN1 with residues of transmembrane segments of neighboring subunits. Our results show that although M4s in NMDARs are not important for receptor assembly and surface expression, the residues at the subunit interface are substantially involved in M4 recognition of the core receptor and regulation of PS efficacy. Because mutations in the M4 of GluN1 specifically resulted in loss of PS-induced inhibition of GluN1/GluN2A and GluN1/GluN3A NMDAR currents, our results point to distinct roles of M4s in NMDAR modulation and highlight the importance of the evolutionarily newly evolved M4 for selective *in vivo* modulation of glutamate- and glycine-activated NMDARs by steroids.

## Introduction

The majority of excitatory activity in the central nervous system (CNS) is mediated by the neurotransmitter glutamate. At postsynaptic neurons, glutamate binds to a wide range of glutamate receptors. Ionotropic glutamate receptors (iGluRs) are one of the major classes of cation-selective ion channels and are divided into four main classes, the AMPA receptors (GluA1-A4), kainate receptors (GluK1-K5), NMDA receptors (GluN1, GluN2A-D, and GluN3A-B), and the delta receptors (GluD1-D2). These receptors are widely distributed in the CNS and play important roles in CNS development, the formation of respiratory and locomotor rhythms, and processes such as learning, memory, and neuroplasticity (Collingridge and Bliss, 1995; Yashiro and Philpot, 2008). iGluRs are characterized by a tetrameric structure of four identical or similar subunits that all follow the same scheme. The subunits are modular and consist of an extracellular amino-terminal domain (ATD), a ligand-binding domain (LBD), the transmembrane domain (TMD), and an intracellular carboxy-terminal domain (CTD). The transmembrane domain consists mainly of the secondary structure motif of α-helices that embed into the cell membrane, anchoring the receptor in the synaptic membrane (Sheng and Pak, 1999). The TMD of iGluRs consists of transmembrane helices M1, M3, and M4 and a membrane loop named M2. The cation-selective pore in the center of the assembled receptor is formed by helices of M1, M2, and M3 (Traynelis et al., 2010). With this pore structure, they inversely resemble bacterial potassium channels, assuming an evolutionary connection (Wo and Oswald, 1995). Thus, it has been shown that it is possible to generate functional chimeras between iGluRs and potassium channels lacking the M4 segment (Schönrock et al., 2019). These findings are also consistent with the functional subunit structure of a bacterial iGluR (i.e., GluR0 from *Synechocystis*) consisting of subunits with only two transmembrane segments (M1 and M3) lacking M4.

The role of the evolutionarily newly formed M4 in receptor function of iGluRs is still unclear (see for example Amin et al., 2017). Structurally, it is distant from the other membrane helices of its own subunit and can only interact with M1 or M3 of the neighboring subunit. For example, in AMPA receptors, there is evidence that the M4 is involved in receptor assembly to the “dimer of dimers” principle. Subunits lacking the M4 segment caused receptors to stack in the endoplasmic reticulum and fail to tetramerize, whereas subunit dimerization appears to function correctly. Single substitutions in the M4 segment at interaction sites near the M1 and M3 segments of adjacent subunits impaired receptor association, suggesting a role for these positions within receptor assembly (Salussolia et al., 2013). For NMDA receptors, there is evidence for M4 involvement in masking retention signals in the M3 of the adjacent subunit within the endoplasmic reticulum, leading to a strong decrease in receptor surface expression in the absence of M4 (Horak et al., 2008). Consistent with this finding, removal of the M4 from GluN1/GluN2A receptors resulted in non-functional receptors (Schorge and Colquhoun, 2003). Moreover, tryptophan screens of the M4 identified different positions at the extracellular end of the M4 that influenced GluN1/GluN2A receptor functionality, whereas positions at the more intracellular end of the M4 segment showed a greater impact on biogenesis and surface expression (Amin et al., 2017). In addition to being involved in NMDAR assembly and function, the M4 appears to be critical for receptor modulation. The neurosteroid pregnenolone sulfate (PS) has a bivalent function at NMDARs that is thought to be mediated by two distinct binding sites. First, PS acts as a positive allosteric modulator (PAM) at NMDARs containing GluN2A or GluN2B subunits, and second, PS acts as a negative allosteric modulator (NAM) at NMDARs containing GluN2C or GluN2D subunits. The PAM binding site of PS is thought to be in the transmembrane region of the GluN2 subunit (Horak et al., 2006; Wilding et al., 2016). Further experiments showed that there are two obligate regions for the effects of PS on all combinations of NMDARs. The J/K helix connecting the LBD to the M4 and the M4 segment itself are required for modulation (Jang et al., 2004). Recent findings through molecular dynamics simulations and alanine screening indicate that the binding site responsible for positive modulation of GluN1/GluN2A/B indeed appears to be localized at the GluN2 M4 interface. These simulations also raise the prospect of another binding site at the appropriate positions for the GluN1 M4 interface that does not carry the PAM effect (Krausova et al., 2020).

In this study, we addressed the question of the extent to which the M4 helix of NMDARs is required for receptor function. To answer this question, we used an approach of M4 truncation, separate M4 segment co-expression, and point mutations in NMDARs to gain insight into the influence of the M4 helix on functionality, assembly, and steroid modulation. To this end, we exploited the ability of separate M4 expression to rescue the functionality of the M4-truncated core receptor. Using this approach, we clearly demonstrate that the M4 helix is not involved in assembly and surface expression but is essential for NMDAR functionality. Furthermore, we found possible attachment sites of the GluN1 M4 to the M1 and M3 of GluN2A that have a strong influence on the rescue effect. In addition, we identified a specific steroid recognition site at the GluN1 M4 that mediates the NAM effect of PS on GluN1/GluN2A and GluN1/GluN3A NMDARs.

## Materials and Methods

Chemicals were purchased from Sigma (Taufkirchen, Germany). Restriction enzymes, Phusion polymerase, and T4 ligase were purchased from Thermo Fisher (Waltham, United States).

### DNA Constructs, Oocyte Expression and Electrophysiology

The GluN1-1a (*rattus norvegicus* P35439), GluN2A (*mus musculus* P35436), GluN2D (*mus musculus* Q03391), and GluN3A (*rattus norvegicus* Q9R1M7) expression constructs in the pNKS2 vector used have been described previously (Honer et al., 1998; Madry et al., 2007b). The M4-deleted GluN1^1–802^ (named GluN1^ΔM4^) and GluN3A^1–893^ (GluN3A^ΔM4^) and the M4 constructs GluN 1^802–925^ (named M4^N1^) and GluN3A^893–1092^ (M4^N3A^) tagged with the respective subunit signal peptide were generated using a Nhe I restriction site. GluN2A^1-803^ (GluN2A^ΔM4^) and GluN2A^804–1464^ (M4^N2A^) constructs were generated by overhang PCR. Biochemical expression analyses were performed using a C-terminal truncated GluN2A^1–929^ construct (GluN2A*) to avoid the confounding effect of the C-terminus during purification steps (Mesic et al., 2016). Point mutations were generated by miss-match PCR. All constructs were confirmed by DNA sequencing (Seqlab, Göttingen, Germany). After plasmid linearization with NotI, cRNA was synthesized using the Amplicap-Max™-SP6 High Yield Message Maker Kit from Cellscript (Madison, Wi, United States) as described by Mesic et al. (2016). Oocytes were obtained from female *Xenopus laevis* after anesthesia with 0.2% tricaine in water after the approval of the Technische Universtät Darmstadt (Agreement V54-19c20/15 DA8/No. 20). Oocytes were isolated and preserved as previously described (Laube et al., 1997). For electrophysiological analysis, the concentration of the cRNA samples were adjusted to 1 μg/μl and oocytes were injected with a volume of 50 nl of the respective wt, M4-deleted, and/or M4 cRNAs in a 1:1:1 ratio. Voltage-clamp recordings of glycine- and glutamate-induced currents in the presence of saturating concentrations of l-glutamate and glycine, respectively, were performed 48–72 h after injection in Mg^2+^-free frog Ringer’s solution containing (mM) 115 NaCl, 1 KCl, 0.9 CaCl_2_ and 10 Hepes (Laube et al., 1998) at a holding potential of −70 mV by two-electrode voltage clamp (TEVC) according to Laube et al. (1997). In brief, TEVC measurements were performed at room temperature using a GeneClamp 500 B amplifier and a Digidata 1322A as an A-D converter. Measurements were recorded using Clampex 10.7 (Molecular Devices, San Jose, United States) at 5 kHz after low-pass filtering at 200 Hz. Microelectrodes with a resistance of 0.8–2.3 MΩ were filled with 3 M KCl. For application, compounds were dissolved in Ringer’s solution, except for pregnenolone sulfate, whose stock solution (100 mM) was prepared in DMSO. For I_max_ current determination of GluN1/GluN2A receptors, glycine and glutamate (100 µM each) were coapplied for 15 s. For dose-response analysis, the respective co-agonist was applied at 100 µM. Measurements of GluN1/GluN3A receptors were made with a 10 s application of 1 mM glycine after preapplication of MDL-29951 (Madry et al., 2007a). For dithiothreitol (DTT) treatments, oocytes were super-fused with 2 mM DTT for 100 s before agonist application in the presence of 2 mM DTT, as described by Lynagh et al. (2013). Pregnenolone sulfate (PS) was always pre-applied 45 s before application of the respective agonists. TEVC recordings were analyzed using Clampfit 10.7 (Axon industries). For dose-response analysis, currents were normalized to the maximum inducible peak-current (I_max_) and fitted with variable slope nonlinear regression in GraphPad Prism 7.0 (GraphPad Software Inc., La Jolla, United States) as previously described (Lynagh and Laube, 2014).

### Labeling, Purification and SDS-PAGE of NMDA Receptor Complexes

For expression analysis, surface receptors were labeled with Pierce™ Premium Grade Sulfo NHS-SS-Biotin (Thermofisher, Waltham, United States) and purified using Streptavidin High Performance Spintrap™ (Sigma-Aldrich, St. Louis, United States). If samples were also treated with DTT, they were incubated with 100 mM DTT (Stocksolution 2 mM DTT in ddH_2_O) for 20 min at 56°C. Isolated surface proteins were separated on linear 10% SDS-PAGE gels. PVDF membranes and the Trans-Blot®Turbo™Blotting System (Biorad, Hercules, United States) were used for Western blot analysis. Two different antibodies were used as primary antibodies, firstly the GluN1-CTD was addressed with an antibody from Merck Millipore (Darmstadt, Germany) diluted 1:1,000 in TBS, and secondly the GluN1-NTD epitope was addressed with an antibody from Alomone Labs (Jerusalem, Israel) 1:500 in TBS. A horseradish peroxidase-labeled secondary antibody (1:20,000 in TBS) detecting mouse or rabbit IgG was used. Immunoreactive bands were visualized with the Pierce™ ECL Western Blotting Substrate (Thermofisher, Waltham, United States) using the ChemiDoc MP Imaging System (Biorad, Hercules, United States). Metabolic labeling with (35S) methionine (0.2 Mbq per oocyte; >40 TBq/mmol, Amersham Biosciences) was performed as previously described (Schüler et al., 2008). Purification of C-terminal His6-tag-labeled GluN1 and GluN1^ΔM4^ by Ni^2+^-NTA agarose (Qiagen) chromatography was performed as in (Madry et al., 2007a). (35S)-Methionine-labeled protein samples were solubilized in SDS sample buffer containing 20 mM dithiothreitol and electrophoresed in parallel with molecular mass markers (SeeBlue^®^ Plus2 Pre-Stained Standard, Invitrogen) on 8% Tricine-SDS polyacrylamide gels. Gels were blotted, fixed, dried, and exposed to BioMax MR films (Kodak, Stuttgart, Germany) at −80°C. The radioactivity of each protein band was quantified using a PhosphorImager (Molecular Dynamics) and analyzed using the ImageQuant software package. Cy5-NHS labeling (Amersham Biosciences) and subsequent SDS-PAGE were performed as described in (Mesic et al., 2016) and scanned with a gel imager (Typhoon 9,400, Amersham Biosciences) as described (Madry et al., 2007a). To distinguish between mature and immature receptor complexes, 10 µl of affinity-purified receptor was incubated in reducing sample buffer (20 mM DTT, 1% (w/v) SDS) with 1% (w/v) octylglucoside containing 5 U endoglycosidase H (Endo H) or peptide: N-glycosidase F (PNGase F; both NEB, Frankfurt, Germany) at 37°C for 1 h, and protein samples were analyzed by SDS-PAGE as described above.

### Sequence and Structural Analysis

Sequence alignments of the GluN1, GluN2, and GluN3 receptor sequences (taken from UniProt) were performed using the Multiple Sequence Alignment Tool from EMBL-EBI (Cambridge, United Kingdom). UCSF Chimera (Pettersen et al., 2004) was used for structural analysis, with the GluN1/GluN2A/GluN2B structure 5UP2 (Lü et al., 2017). The distances between the Cα-atoms of the amino acids were determined using the distance tool in Chimera. Images were generated using PyMOL 1.2 (http://www.pymol.org).

### Statistical Analysis

Statistical analysis was performed using GraphPad Prism 7.0 (GraphPad Software Inc., La Jolla, United States). A Gaussian distribution was assumed for the values obtained. Paired or unpaired Student’s t-test was used to determine significances. Data in Figures 3E,G and Figure 4B,D were analyzed by one-way ANOVA followed by Dunnett´s multiple comparisons test. *p* < 0.05 (*), *p* < 0.01 (**), *p* < 0.001 (***), and *p* < 0.0001 (****). Values shown represent mean ± SEM.

## Results

### M4 Is Essential for the Function of Glutamate-Gated GluN1/GluN2A NMDA Receptors

**FIGURE 1:**
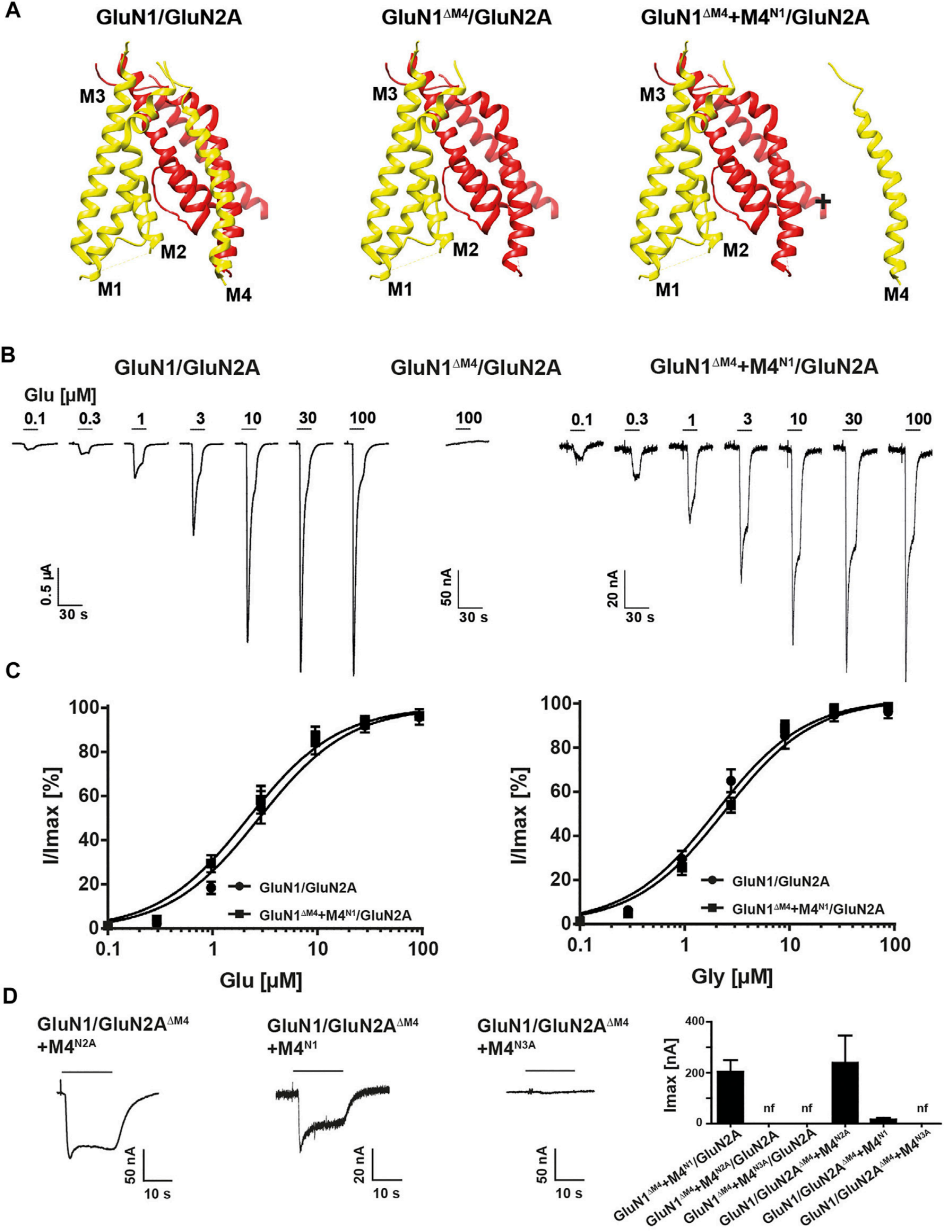
Functional characterization of M4-deleted and M4-co-expressed GluN1/GluN2A NMDA receptors. **(A)** Structure of the TMD region of a GluN1 (yellow)/GluN2A (red) NMDAR showing the peripheral localization of the M4 compared to M1, M2 and M3 of its own subunit. Structure in the middle and right show the M4-truncated receptor (GluN1^ΔM4^/GluN2A) and exemplary M4-truncated receptor with the separate M4 segment (GluN1^ΔM4^+M4^N1^/GluN2A). Structural analysis was performed from PDB 5UP2 using UCSF Chimera. **(B)** Agonist response properties of wt GluN1/GluN2A receptor (left), nonfunctional truncated GluN1^ΔM4^/GluN2A (middle), and GluN1^ΔM4^+M4^N1^/GluN2A receptor (right) rescued by M4 segment co-expression recorded by superfusion of increasing concentrations of glutamate in the presence of glycine 2–3 days after injection. **(C)** Glutamate or glycine dose response curves for wt GluN1/GluN2A and GluN1^ΔM4^+M4^N1^/GluN2A NMDARs in the presence of glycine (left) or in the presence of glutamate (right). Data represent mean ± SEM values. **(D)** Representative TEVC recordings of GluN1/GluN2A^ΔM4^+M4^N2A^ (left) and GluN1/GluN2A^ΔM4^+M4^N1^ (middle) show rescued functionality of GluN2A-M4 truncated receptors with M4^N1^ or M4^N2A^. No measurable currents could be obtained for GluN1/GluN2A^ΔM4^+M4^N3A^ (right), highlighting the differences between the M4 segments of GluN1, GluN2A, and GluN3A. Right panel shows I_max_ currents of co-expression of M4-deleted GluN1/GluN2A NMDARs in the presence of M4 fragments (*n* = 5–12). Data represent mean values ± SEM.

To analyze the function of the M4 in NMDARs, we first examined the functional properties of the glycine-binding GluN1 subunit truncated by the M4 helix (GluN1^ΔM4^; for illustration, see Figure 1A and MatMet) after co-expression with the glutamate-binding wild-type (wt) GluN2A subunit. In contrast to wt GluN1/GluN2A receptors, no currents could be measured in *Xenopus laevis* oocytes expressing GluN1^ΔM4^/GluN2A subunits after application of saturating concentrations of glutamate and glycine by two-electrode voltage clamp (TEVC) (Figure 1B). However, co-expression of GluN1^ΔM4^/GluN2A receptors in the presence of a protein fragment containing the M4 of GluN1 (M4^N1^) resulted in agonist-induced currents (Figure 1B). To investigate whether M4 protein fragments from other NMDAR subunits would also rescue GluN1^ΔM4^/GluN2A receptor function, we co-expressed homologous constructs of the M4 of GluN2A (M4^N2A^) and GluN3A (M4^N3A^) subunits. For both M4 fragments, no detectable currents were obtained in GluN1^ΔM4^/GluN2A receptors upon co-expression, indicating a unique role of the M4^N1^ segment in the rescue of GluN1^ΔM4^/GluN2A receptors (see Figure 1D). To analyze the pharmacological properties of GluN1^ΔM4^/GluN2A receptors in the presence of the M4^N1^ protein fragment, the maximum inducible currents (I_max_) and the respective EC_50_ values of agonists were measured in GluN1/GluN2A and GluN1^ΔM4^+M4^N1^/GluN2A receptors. GluN1^ΔM4^+M4^N1^/GluN2A-expressing oocytes showed lower I_max_ than GluN1/GluN2A receptors upon application of saturating concentrations of glutamate and glycine (GluN1/GluN2A: 2.5 ± 0.4 µA, vs GluN1^∆M4^+M4^N1^/GluN2A: 0.2 ± 0.05 µA, t (26) = 10.79; *p* < 0.0001; Figures 1B,D). In contrast, EC_50_ values of glutamate and glycine affinities were not changed compared to wt GluN1/GluN2A receptor (glutamate: 2.8 ± 0.3 µM vs 2.3 ± 0.3 µM; glycine: 2.1 ± 0.2 µM vs 2.4 ± 0.4 µM for GluN1/GluN2A and GluN1^∆M4^+M4^N1^/GluN2A receptors, respectively; Glu: t (12) = 1.6; *p* = 0.42 and Gly: t (14) = 0.92; *p* = 0.37 see Figure 1C). In conclusion, only co-expression of the M4 fragment of the GluN1 subunit rescued GluN1^ΔM4^/GluN2A channel function without altering glutamate and glycine EC_50_ values.

To analyze the role of the M4 of GluN2 subunits in GluN1/GluN2 receptor function, we examined the functional properties of the glutamate-binding GluN2A subunit (GluN2A^ΔM4^) truncated by the M4 segment after co-expression with the GluN1 subunit. Again, only the presence of the M4 protein fragment of GluN2A (M4^N2A^) resulted in inducible currents in GluN1/GluN2A^ΔM4^ receptor-expressing oocytes with an I_max_ of 0.24 ± 0.1 µA (n = 5; Figure 1D). In contrast to our results with the GluN1^∆M4^ construct, where only the M4 of the GluN1 subunit rescued channel function, surprisingly, co-expression of the M4 of the GluN1 subunit (M4^N1^) also rescued receptor function of the GluN2A^ΔM4^ construct (I_max_ 0.016 ± 0.007 µA, n = 5; Figure 1D). However, co-expression of the M4^N3A^ fragment with GluN1/GluN2A^ΔM4^ receptors showed no rescue effect (Figure 1D). Thus, both the M4 of GluN1 and of GluN2A could rescue the channel function of GluN1/GluN2A^ΔM4^ receptors after co-expression. In another experiment, we now wanted to test whether the function of both M4-deleted GluN1 and GluN2A subunits could be rescued by co-expression with an M4 fragment. However, the functionality of the GluN1^ΔM4^/GluN2A^ΔM4^ receptors could not be restored after co-expression with either the M4^N1^, M4^N2A^ or in combination with both (data not shown). In summary, our analyses of the rescue of M4-truncated GluN1/GluN2A NMDA receptors by separately expressed M4 fragments indicate that 1) specific recognition interactions must exist between the M4 and the core receptor, since only in the presence of selected NMDAR-M4 fragments the deleted GluN1/GluN2A receptors were functional, and 2) the basic pharmacological properties were not altered, since the apparent glutamate and glycine affinities (EC_50_ values) remained the same compared with wt GluN1/GluN2A.

### M4-Deleted NMDA Receptor Subunits Retain Receptor Surface Expression

**FIGURE 2:**
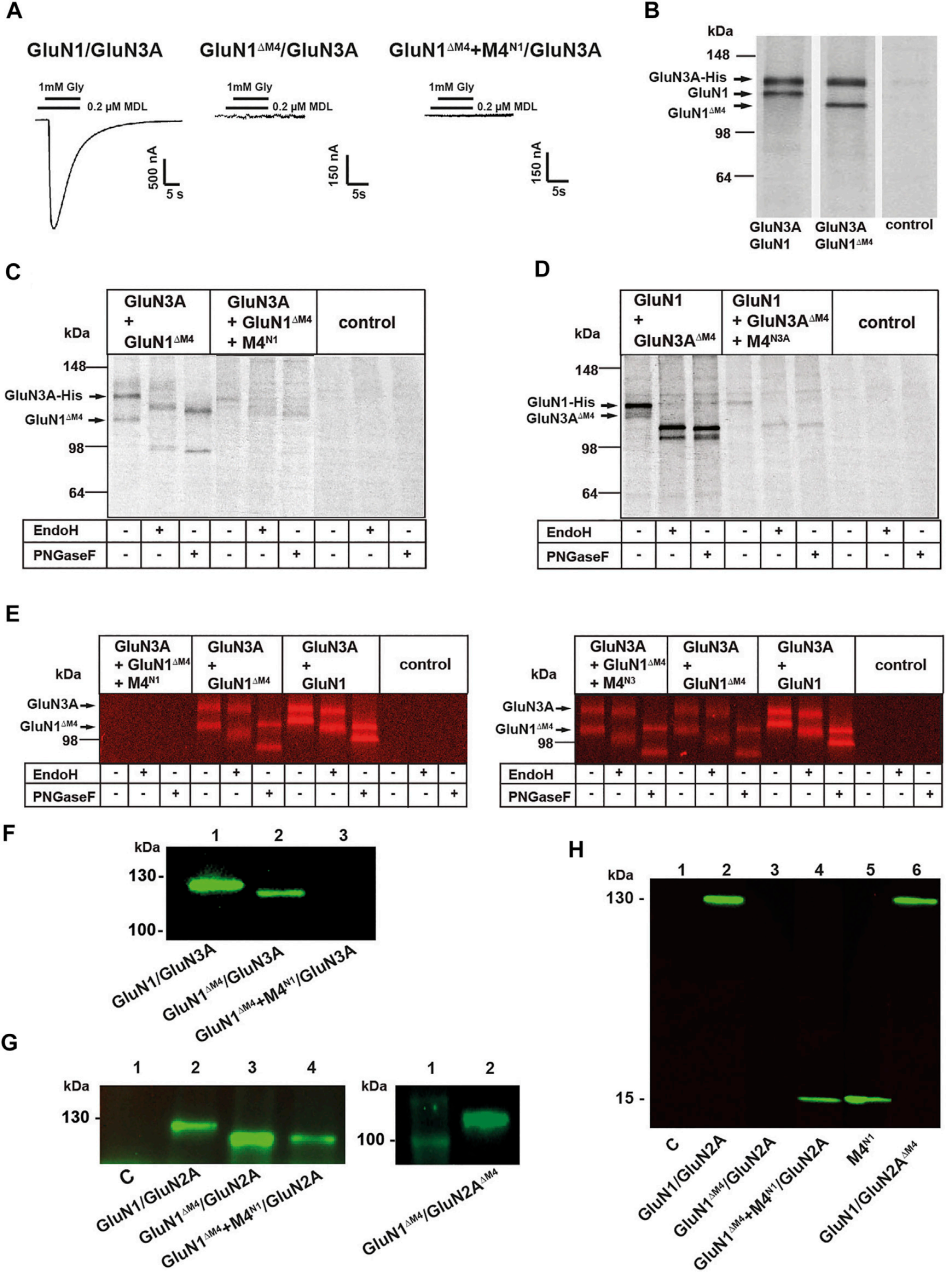
Influence of M4 on the surface expression of NMDARs. **(A)** Representative images of GluN1/GluN3A, nonfunctional GluN1^ΔM4^/GluN3A, and GluN1^ΔM4^+M4^N1^/GluN3A receptor combinations. M4^N1^ failed to rescue functionality in M4-truncated GluN1/GluN3A receptors. **(B)** SDS-PAGE of metabolic (35S) methionine-tagged GluN1/GluN3A and GluN1^ΔM4^/GluN3A receptors with simultaneous purification of C-terminal His-tagged receptor subunit constructs by metal affinity chromatography (His-tag purification applies to B–E.). Both the GluN1/GluN3A and GluN1^ΔM4^/GluN3A receptors were correctly expressed. **(C, D)** SDS-PAGE of metabolic (35S) methionine-labeled GluN1/GluN3A receptor combinations. Glycosylation status was determined by PNGaseF or EndoH treatment and showed that GluN1^ΔM4^/GluN3A and GluN1/GluN3A^ΔM4^ were N-glycosylated, indicating cell surface localization. Co-expression of the respective M4 segment resulted in impaired receptor expression or biogenesis in the ER. **(E)** SDS-PAGE of Cy5 surface-stained GluN1/GluN3A and GluN1^ΔM4^/GluN3A and GluN1^ΔM4^+M4^N1^/GluN3A receptors (left) and GluN1/GluN3A and GluN1^ΔM4^/GluN3A and GluN1^ΔM4^+M4^N3A^/GluN3A receptors (right) purified *via* His-tag with/without PNGase F or EndoH treatment. Only coexpression of the M4^N1^ segment resulted in loss of surface expression. **(F–H)** SDS-PAGE was performed with the surface proteins isolated by biotin affinity purification. Western blot performed with a primary antibody against the GluN1-NTD shows surface-expressed GluN1/GluN3A and GluN1^ΔM4^/GluN3A receptors and a complete loss of surface expression for the GluN1^ΔM4^+M4^N1^/GluN3A co-expressed with the M4 segment. **(G)** Western blot performed with a primary antibody against the GluN1-NTD shows correct surface expression for GluN1/GluN2A, GluN1^ΔM4^/GluN2A, and GluN1^ΔM4^+M4^N1^/GluN2A without affecting M4 segment co-expression (left) and for GluN1^ΔM4^/GluN2A^ΔM4^ (right). **(H)** Western blot analysis using a primary antibody against the GluN1 CTD showing that the M4 segment was well expressed at the cell surface when GluN1^ΔM4^+M4^N1^/GluN2A and M4^N1^ were expressed alone. The GluN1/GluN2^ΔM4^ receptor was also well expressed at the surface.

To investigate whether M4-truncated glycine-gated GluN1/GluN3 NMDARs are also rescued in function by M4 fragments, we analyzed our GluN1^ΔM4^ with wt GluN3A subunit in the absence and presence of M4^N1^. In contrast to our results with the M4-truncated GluN1/GluN2A receptors, neither application of the agonist glycine (1 mM) alone nor in combination with the potentiating ligand MDL-29951 (0.2 µM, see Madry et al., 2007a) caused detectable currents in GluN1^ΔM4^/GluN3A receptors in the presence of M4^N1^ (Figure 2A). To analyze whether the GluN1^ΔM4^ assembles with the GluN3A subunit, we performed SDS-PAGE after metal affinity chromatography with a C-terminal hexahistidyl-tagged GluN3A subunit (GluN3A-His) after metabolic (35S) methionine labeling. Co-expression of GluN3A-His with either the GluN1 or the GluN1^ΔM4^ subunit resulted in two (35S) methionine-labeled subunits each with similar 1:1 intensities (Figure 2B and data not shown) after autoradiographic analysis of the radioactive bands based on the total number of methionine residues per subunit (30 per GluN1, 25 per GluN1^ΔM4^, and 33 per GluN3A; see Mesic et al.,. 2016). This indicated an unchanged assembly behavior of the GluN1^ΔM4^/GluN3A receptor. To understand the differential rescue effect of the M4^N1^ fragment in GluN1^ΔM4^/GluN2A and GluN1^ΔM4^/GluN3A receptor function, we examined the expression pattern of the GluN1^ΔM4^ and GluN3A-His subunits after co-expression with the M4^N1^ fragment (Figure 2C). In contrast to the result with the GluN1^ΔM4^/GluN3A receptor in the absence of M4^N1^, surprisingly, both the GluN3A and GluN1^ΔM4^ subunits showed a marked reduction in total protein amount after co-expression with the M4^N1^ fragment (compare lanes 1 and 4 in Figure 2C). We therefore performed the reverse experiment and analyzed the biochemical and functional properties of the M4-truncated GluN3A subunit (GluN3A^ΔM4^) after co-expression with the wt-GluN1 subunit in the absence and presence of the M4^N3A^ fragment. Similar to GluN1^ΔM4^/GluN3A receptors, co-expression of GluN1/GluN3A^ΔM4^ with the M4^N3A^ fragment resulted in nonfunctional channels and a comparable decrease in protein expression of GluN1/GluN3A^ΔM4^ receptors (see Figure 2D compared with 2C). These data suggest that the respective M4 fragments impede protein expression of GluN1^ΔM4^/GluN3A and GluN1/GluN3A^ΔM4^ receptors, respectively.

Interestingly, however, we noticed a differential shift in the molecular masses of the GluN1^ΔM4^/GluN3A and GluN1/GluN3A^ΔM4^ receptor proteins upon treatment with PNGase F and EndoH (see Figures 2C,D, lanes 2 and 3), indicating a putative surface expression of the proteins in the absence of M4. We therefore performed surface labeling experiments with affinity purification of GluN1^ΔM4^/GluN3A-His receptors from Cy5 surface-labeled oocytes using a Cy5-NHS ester-based protocol (Schüler et al., 2008). Purification of GluN1^ΔM4^/GluN3A receptors confirmed our assumption that the GluN1^ΔM4^ and GluN3A subunits, just like GluN1/GluN3A receptors, assemble properly and are efficiently located at the cell surface (Figure 2E, lanes 5, 6 and lanes 8, 9). Consistent with our results with the (35S) methionine-labeled subunits (see Figure 2C), analysis of the surface-labeled GluN1^ΔM4^/GluN3A receptors showed also a complete loss of surface expression for GluN1^ΔM4^/GluN3A receptors after co-expression with the M4^N1^ fragment (Figure 2E, lanes 1–3). We therefore planned further surface labeling experiments to investigate whether the difference in rescue of GluN1^ΔM4^/GluN3A and GluN1^ΔM4^/GluN2A channel function in the presence of the M4^N1^ fragment could be explained by a loss of plasma membrane insertion of the GluN1^ΔM4^/GluN3A surface receptors. Interestingly, analysis of surface-labeled GluN1^ΔM4^/GluN3A receptors in the presence of the M4^N3A^ fragment showed no loss of surface expression for GluN1^ΔM4^/GluN3A receptors (Figure 2E right panel, lanes 1–3), again highlighting differences between the M4 segments of GluN1, GluN2A, and GluN3A.

We therefore planned further surface labeling experiments to investigate whether the difference in rescue of GluN1^ΔM4^/GluN3A and GluN1^ΔM4^/GluN2A channel function in the presence of the M4^N1^ fragment could be explained by a loss of plasma membrane insertion of the GluN1^ΔM4^/GluN3A surface receptors. Thus, we performed cell surface biotinylation of GluN1^ΔM4^/GluN3A- and GluN1^ΔM4^/GluN2A receptor-expressing oocytes, followed by separation of the nonbiotinylated intracellular proteins using a streptavidin agarose pull-down approach. Western blot analysis of the bound biotinylated protein fraction with a primary antibody against the GluN1 extracellular epitope revealed specific bands for GluN1 (125 kDa) and GluN1^ΔM4^ (110 kDa) subunits after expression with the GluN3A and GluN2A subunits, respectively (Figures 2F,G). Interestingly, GluN1^ΔM4^ protein was absent at the surface when expressed in GluN1^ΔM4^/GluN3A receptors in the presence of the M4^N1^ fragment (Figure 2F), whereas surface-tagged GluN1^ΔM4^ protein was found when co-expressed in GluN1^ΔM4^/GluN2A receptors with the M4^N1^ fragment (Figure 2G). Metabolic labeling of a GluN2A*-His construct (see Material and Methods) and the GluN1^ΔM4^ subunit in the absence and presence of M4^N1^ showed no difference in the [35S]methionine-labeled subunit bands and no remarkable decrease in total protein concentrations (see Supplementary Figure S1. data). This is in marked contrast to our results obtained with the expressed GluN1^ΔM4^/GluN3A subunits, where expression was abolished in the presence of M4^N1^ (Figure 2C). Apparently, in contrast to the GluN1^ΔM4^/GluN3A- and GluN1/GluN3A^ΔM4^-receptors, the respective co-expressed M4 fragments did not interfere with the surface expression/assembly of the GluN1^ΔM4^/GluN2A- or GluN1/GluN2A^ΔM4^-receptor complex.

To verify whether possibly the absence of all M4 segments in GluN1/GluN2A receptors would affect surface expression, we performed cell surface biotinylation of GluN1^ΔM4^/GluN2A^ΔM4^ receptor-expressing oocytes. Western blot analysis of the bound biotinylated protein fraction with the primary antibody against the extracellular GluN1 epitope revealed a specific band for the GluN1^ΔM4^ (110 kDa) subunit after expression with the GluN2A^ΔM4^ subunit (Figure 2G right), suggesting that the M4 segments of the NMDAR are not important for surface expression. We therefore hypothesized that the M4-deleted receptors might interact with separately-expressed M4 fragments in the plasma membrane, where they are converted into functional receptors. Therefore, we performed cell surface biotinylation of GluN1^ΔM4^/GluN2A receptor-expressing oocytes with M4^N1^, followed by Western blot analysis of the bound biotinylated protein fraction using the primary antibody against the C-terminal (CTD) epitope of GluN1. Using this specific CTD antibody, we detected a protein band of 15 kDa representing our M4^N1^ fragment (Figure 2H). Interestingly, surface expression of this M4N1 protein was observed not only in GluN1^ΔM4^/GluN2A-expressing oocytes but indeed also during single-cell expression (Figure 2H), supporting the possibility that our M4 constructs might assemble with M4-deleted GluN1^ΔM4^/GluN2A or GluN1/GluN2A^ΔM4^ receptors in the cell membrane to form functional receptors. In contrast, for GluN^1ΔM4^/GluN3A- or GluN1/GluN3A^ΔM4^-receptors, interaction of M4s with the core receptor would lead to early degradation of the receptor protein. However, further experiments would be needed to confirm these assumptions.

Thus, our analyses of surface-labeled M4-deleted NMDARs demonstrate that both M4-deleted glutamate-gated GluN1/GluN2A and glycine-gated GluN1/GluN3A receptors, although nonfunctional, localize efficiently to the cell surface. The differences in the restoration of channel function for GluN1^ΔM4^/GluN2A- and GluN1/GluN2A^ΔM4^-receptors compared to GluN1^ΔM4^/GluN3A- and GluN1/GluN3A^ΔM4^-receptors by selected M4 fragments indicate that 1) our M4 constructs selectively interact with core receptors but 2) apparently differentially affect the biogenesis of the respective GluN1/GluN2A and GluN1/GluN3A core receptor. Thus, the corresponding M4 apparently leads to loss of expression of M4-truncated GluN1/GluN3A receptors by degradation whereas surface localization of singly expressed M4 fragments could possibly lead to functional channels by interaction with M4-deleted GluN1/GluN2A surface receptors. However, in summary, M4-deleted GluN1/GluN2A and GluN1/GluN3A receptors are correctly, but non-functionally, aligned in the plasma membrane, but only co-expression of the corresponding M4 fragment in M4-deleted GluN1/GluN2A receptors can rescue channel function by interaction with the core receptor.

### Mapping of a Functional M4 Interface of the GluN1 Subunit in GluN1/GluN2A Receptors

**FIGURE 3:**
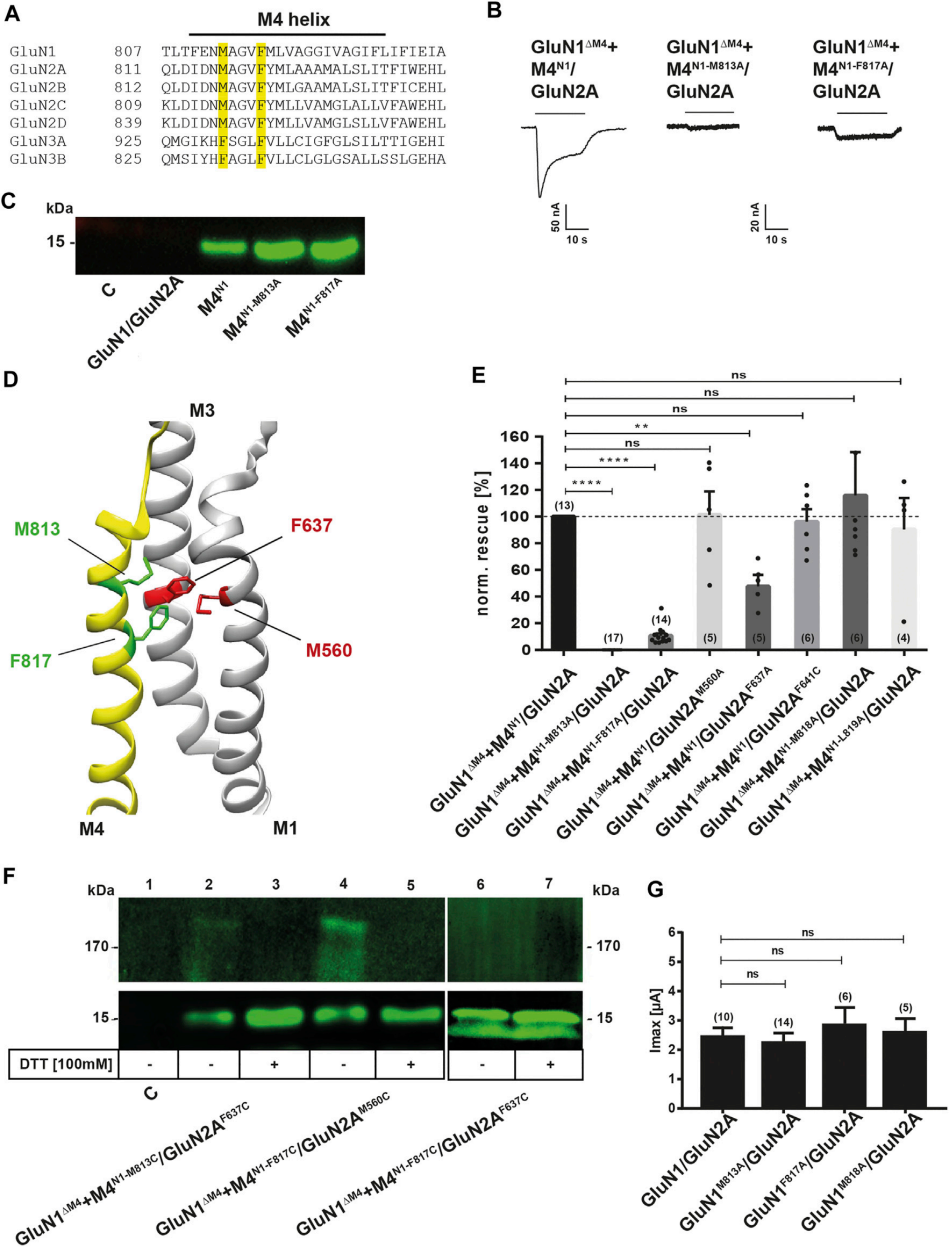
Examination of the possible interaction residues of GluN1-M4 and GluN2A-M1/M3. **(A)** Sequence alignment of the human GluN1, GluN2 and GluN3 M4 segments. Sequences were taken from Uniprot and sequence alignment was performed using the Multiple Sequence Alignment Tool from EMBL-EBI. The residues identified as possible attachment sites, M813 and F817, are highlighted in yellow. **(B)** Representative TEVC images of GluN1^ΔM4^+M4^N1^/GluN2A, GluN1^ΔM4^+M4^N1-M813A^/GluN2A, and GluN1^ΔM4^+M4^N1-F817A^/GluN2A show the impaired rescue effect of the two point mutations. M4^N1-M813A^ resulted in a complete loss of rescue effect, while M4^N1-F817A^ resulted in a ∼90% decrease in rescued current. **(C)** Western blot of surface proteins isolated by biotinylation, with a primary antibody against the GluN1 CTD, showing that M4^N1^, M4^N1-M813A^, and M4^N1-F817A^ are all well expressed at the cell surface, ruling out that impaired expression is responsible for the loss of the rescue effect. **(D)** Representation of GluN1-M4 (yellow) and GluN2A-M1/M3 (gray). Structural analysis was performed using UCSF Chimera with the GluN1/GluN2A structure 5UP2. Amino acids M813 and F817 are colored green, and potential interaction partners M1-M560 (partner of F817) and M3-F637 (partner of M813) are highlighted in red. **(E)** Quantification of the rescue effect of alanine substitutions normalized to the unmutated M4 segment in co-expressed M4^N1^ showed that for M4 mutations M818A and L819A, in contrast to M4^N1-M813A^ and M4^N1-F817A^, there was no effect on the rescued currents. The GluN2A^M560A^ mutation also showed no effect on the rescue effect, suggesting a stronger influence of the F817-M560 interaction. GluN2A^F637A^ showed a strong decrease in rescued I_max_ currents by ∼50%, highlighting the importance of the M813-F637 interaction for M4 binding to the truncated core receptor. Data were analyzed by one-way ANOVA followed by Dunnett´s multiple comparisons test. Number of experiments is given in brackets. **(F)** Western blot of isolated surface proteins, with a primary antibody against the GluN1-CTD. Samples were equally divided and loaded untreated and DTT-treated. The potential interaction partners M813-F637 and F817-M560 were mutated to cysteines to confirm close proximity. GluN1^ΔM4^+M4^N1-M813C^/GluN2A^F637C^ and GluN1^ΔM4^+M4^N1-F817C^/GluN2A^M560C^ both showed both a ∼180 kDa and a ∼15 kDa band under denaturing conditions without DTT. In both cases, the 180 kDa band disappeared after DTT treatment with an increase in the 15 kDa band, indicating a disulfide bond between the M4^M813C^ (or F817C) with the GluN2A^F637C^ (or M560C). The GluN1^ΔM4^+M4^N1-F817C^/GluN2A^F637C^ sample loaded as a control did not result in a band around the 180 kDa mark, indicating that no disulfide bond was formed here. **(G)** Quantification of I_max_ currents of wt- and alanine-substituted full-length GluN1/GluN2A receptors showing no significant decrease, indicating that the M813A, F817A, and M818A mutations do not affect receptor activity in the full-length receptor. Data were analyzed by one-way ANOVA followed by Dunnett´s multiple comparisons test. Number of experiments is given in brackets. Data represent mean ± SEM values.

Taken together, our previous data highlight a particular specificity of M4 transmembrane segment interactions for restoring receptor function. Because co-expression of the M4^N3A^ fragment, in contrast to the M4^N1^ and M4^N2A^ fragments, failed to restore the function of M4-deleted GluN1/GluN2A NMDARs, we interpreted this result as strong evidence for the presence of specific interactions with the M4 fragment, likely mediated by residues that are 1) highly conserved in the GluN1 and GluN2 M4s or 2) are not conservatively exchanged in the GluN3-M4s. Therefore, to further investigate the molecular basis of M4 interaction in GluN1/GluN2A receptors, we identified, based on the published structure of the transmembrane domains of the tetrameric GluN1/GluN2A/GluN2B receptor (Lü et al., 2017; Protein Data Bank entry 5UP2) and their C distance from the M1 or M3 of the adjacent GluN2A subunit, residues M813, F817, V820, I824, G827, and E834 in the M4 of GluN1 targeted for interactions with the core receptor. Through previous mutation studies (Lemke et al., 2016; Amin et al., 2017), it is known that interface mutations starting at position 827 at the cytoplasmic end of GluN1-M4 extremely affect GluN1/GluN2 receptor function and surface expression, whereas interface mutations in the N-terminal region of M4 hardly affect biogenesis. Of these N-terminal interface residues, only residues M813 and F817 are conserved in GluN1 and GluN2 subunits, with methionine 813 replaced by a phenylalanine in GluN3 (see Figure 3A). Thus, only these N-terminal residues M813 and F817, which were likely to be functional after substitution, were eligible for our functional rescue approach with M4 fragments. Co-expression of the M4^N1-M813A^ fragment with GluN1^ΔM4^/GluN2A receptors showed a rescue effect, but it was much smaller compared with wt M4^N1^ (Figures 3B,E). We then substituted phenylalanine 817 to alanine, which is conserved in all NMDAR subunits, to see if this residue also affected rescue of function. The M4^N1-F817A^ mutation similarly resulted in reduced rescue of channel function when co-expressed with the GluN1^ΔM4^/GluN2A receptor Figures 3B,E), suggesting that an interaction of the two residues with the core receptor was likely. As a control, we analyzed the substitutions of methionine 818 (tyrosine in GluN2 and valine in GluN3) in M4^N1-M818A^ and leucine 819 (methionine in GluN2 but also valine in GluN3) in M4^N1-L819A^, which, based on the structure of Lü et al., 2017, were not considered to interact directly with the core receptor. Both mutated M4^N1^ fragments showed a similar rescue effect as wt M4^N1^ after coexpression with the GluN1^ΔM4^/GluN2A receptor (Figure 3E), which strengthened our hypothesis that both residues M813A and F817A might be impaired interactions with the core receptor. However, to rule out the possibility that differences in surface expression of the M4^N1^, M4^N1-M813A^, and M4^N1-F817A^ fragments were the cause of differential rescue of GluN1^ΔM4^/GluN2A channel function, we performed cell surface biotinylation followed by Western blot analysis. For all three M4^N1^ constructs, we detected protein bands of 15 kDa with similar intensities (Figure 3C). This shows that all our M4^N1^ mutants are efficiently expressed and transported to the cell surface, which suggested that substitution of methionine 813 and phenylalanine 817 specifically affects the interaction of M4 with the core receptor. To verify the role of M4^N1-M813^ in GluN1/GluN2A receptors, we mutated residues phenylalanine 637 in M3 of GluN2A (conserved in all GluN2 subunits) and methionine 560 in M1 of GluN2A, which, based on analysis of structures with a Cα distance in the range of 6 Å, were candidates for direct interactions (Figure 3D). Co-expression of M4^N1^ in GluN1^ΔM4^/GluN2A^F637A^ receptors again resulted in impaired functional rescue (Figure 3E), indicating a specific GluN1-M813 GluN2A-F637 interaction, because rescue was not decreased in GluN1^ΔM4^/GluN2A^M560A^ receptors. Similarly, co-expression of the mutation of phenylalanine 641 to alanine in M3 of GluN2A, a residue not located near M813 according to structural analysis, resulted in complete functional rescue (Figure 3E).

To test whether the GluN1-M813 position is indeed close to the amino acid GluN2A-F637 in our M4 fragment rescue experiments, we replaced these residues with cysteines and generated M4^N1-M813C^ and GluN2A^F637C^ constructs. To verify this interaction, we performed cell surface biotinylation of GluN1^ΔM4^/GluN2A^F637C^ with M4^N1-M813C^-expressing oocytes and Western blot analysis with the primary antibody against the C-terminal epitope of GluN1 under denaturing conditions in the absence of dithiothreitol (DTT). Both a ∼180 and a 15-kDa band were detected, indicating the disulfide-linked M4^N1-M813C^/GluN2AF637C and single M4N1-M813C fragments expressed at the cell surface, respectively (Figure 3F). The 180-kDa band decreased in the presence of DTT, whereas the intensity of the 15-kDa band was significantly increased (Figure 3F). This suggests that in GluN1^ΔM4^+M4^N1-M813C^/GluN2A^F637C^ receptors, the introduced cysteines form a disulfide bond that can be released in the presence of DTT, resulting in the loss of the 180-kDa band of the disulfide-bonded M4^N1-M813C^ fragment to GluN2A^F637C^. Because the M4^N1-F817A^ fragment also showed reduced recovery of functionality when co-expressed with GluN1^ΔM4^/GluN2A receptors and is also localized to the TMD interface of GluN1/GluN2A receptors (see Protein Data Bank crystal structure entry 5UP2), we also treated oocytes with DTT expressing the M4^N1-F817C^ and GluN2A^M560C^ mutant, a residue localized at the M4/M1 interface of GluN1 and GluN2A subunits, with a Cα distance of 6Å from F817 (Figure 3D). Similar to GluN1^ΔM4^+M4^N1-M813C^/GluN2A^F637C^ receptors, a ∼180 kDa band decreased in the presence of DTT, whereas the intensity of the 15 kDa band increased again (Figure 3F). In contrast, co-expression of M4^N1-F817C^ with GluN1^ΔM4^/GluN2A^F637C^ did not result in disulfide bond formation (Figure 3F lanes 6, 7). Thus, the GluN1-M813/GluN2A-F637 and GluN1-F817/GluN2A-M560 positions are capable of forming a specific disulfide bond, suggesting that the Cα atoms of the GluN1-M813 and GluN2A-F637 or GluN1-F817 and GluN2A-M560 positions are separated by only about 6 Å. However, co-expression of both GluN1^ΔM4^/GluN2A^F637C^ with M4^N1-M813C^ and GluN1^ΔM4^/GluN2A^M560C^ with M4^N1-F817C^ showed no current responses to saturating concentrations of agonists in both the absence and presence of DTT (*n* = 10) (data not shown). This suggests that the disulfide bonds between transmembrane helices may not be resolved after DTT treatment.

To gain insight into the functional role of interface residues in the full-length GluN1/GluN2A receptor, we mutated residues M813, F817, M818, V820, G827, and E834 in the M4 of GluN1, F637 and F641 in the M3, and M560 in the M1 of GluN2A to alanine, respectively, to analyze the functional properties of the mutants GluN1^M813A^, GluN1^F817A^, GluN1^M818A^, GluN1^V820A^, GluN1^G827A^, GluN1^E834A^, GluN2A^M560A^, GluN2A^F637A^, and GluN2A^F641A^ after co-expression with the respective wt subunit. Interestingly, all N-terminal mutants showed Imax currents and glutamate EC_50_ values comparable to wt-GluN1/GluN2A receptors (Figure 3G and see Table 1). Only the C-terminal mutation G827A and E834A in the M4 of GluN1 were nonfunctional. In conclusion, the residues identified here with the separately-expressed M4 fragment of GluN1 possess a remarkable role in the functional rescue of M4-deleted GluN1/GluN2A receptors, whereas only a minor effect of the point mutations on function within full-length receptors was detected.

**TABLE 1:** I_max_ and EC_50_ values of recombinant wild type and mutant GluN1/GluN2A NMDARs.

Subunit composition	*I*_max_ [µA]	*EC*_*50*_ *glutamate* [µM]
GluN1/GluN2A	2.5 ± 0.3 (10)	2.8 ± 0.3 (7)
GluN1M813A/GluN2A	2.2 ± 0.3 (14)	6.5 ± 1.2 (11)
GluN1F817A/GluN2A	2.7 ± 0.6 (6)	1.7 ± 0.2 (3)
GluN1M818A/GluN2A	2.6 ± 0.5 (5)	2.3 ± 0.4 (4)
GluN1I824A/GluN2A	2.8 ± 0.9 (3)	1.7 ± 0.3 (3)
GluN1G827A/GluN2A	nf (8)	—
GluN1E834A/GluN2A	nf (8)	—
GluN1/GluN2AF637A	2.8 ± 0.1 (3)	3.4 ± 1.8 (4)
GluN1/GluN2AF641A	3.0 ± 0.9 (3)	2.3 ± 0.7 (4)

Values represent mean ± SEM. Numbers of experiments given in parentheses.

### Role of the GluN1-M4 Interfaces in the Action of Pregnenolone Sulfate

**FIGURE 4:**
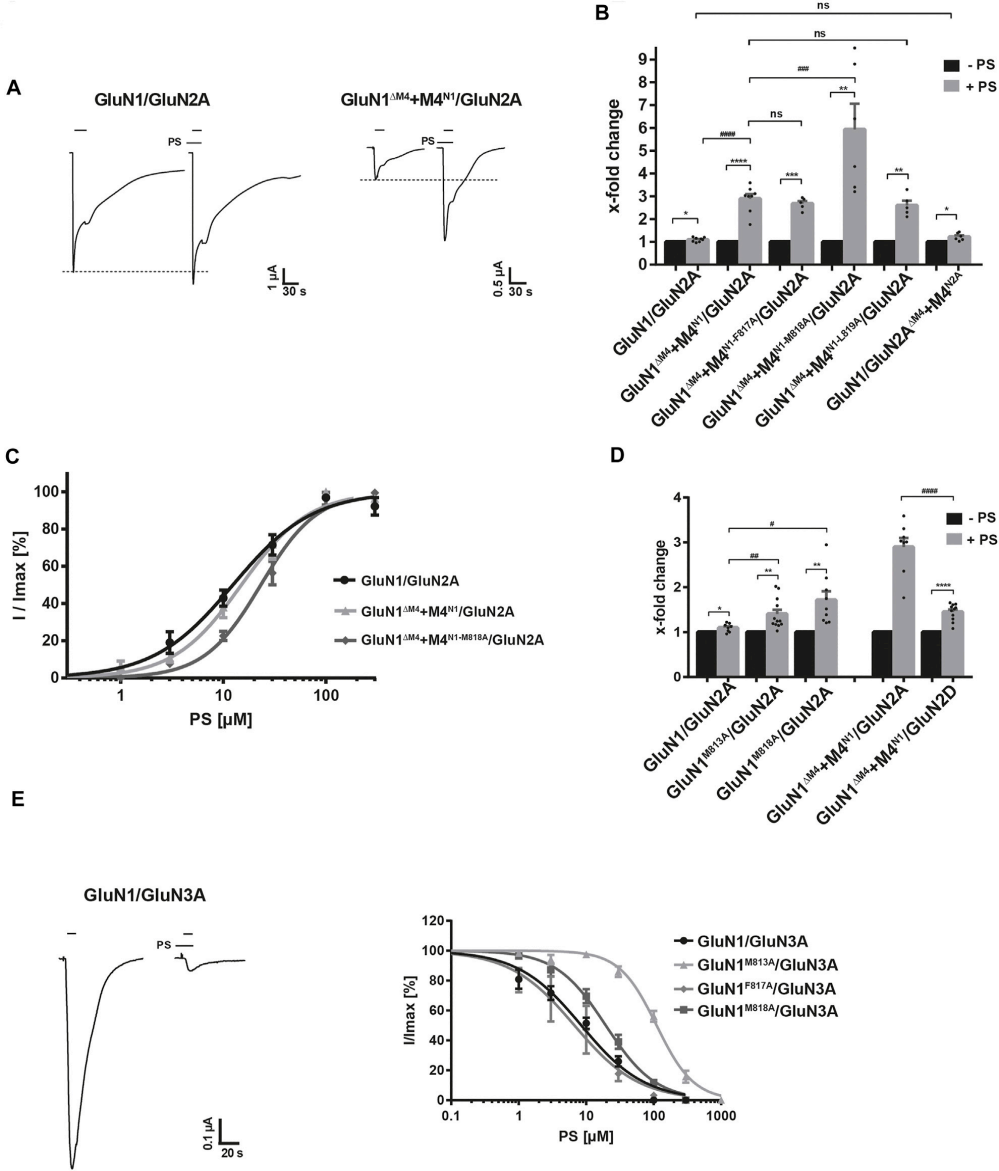
Identification of a NAM recognition site of pregnenolone sulfate at GluN1-M4. **(A)** Representative TEVC images of I_max_ potentiation of PS at GluN1/GluN2A and GluN1^ΔM4^+M4^N1^/GluN2A receptors showing a strong increase in potentiating effect for M4 segment co-expression. **(B)** Quantification of I_max_ potentiation of PS at GluN1/GluN2A, GluN1^ΔM4^+M4^N1^/GluN2A, M4 segment mutants F817A, M818A, and L819A, and GluN1/GluN2A^ΔM4^+M4^N2A^. Coexpression of M4^N1-F817A^ and M4^N1-L819A^ showed no effect on the potentiating effect compared with unmutated M4^N1^. Co-expression of M4^N1-M818A^ showed a strong increase in I_max_ potentiation compared to GluN1^ΔM4^+M4^N1^/GluN2A [t (12) = 3.08; *p* = 0.0095], indicating an impaired NAM recognition site. GluN1/GluN2A^ΔM4^+M4^N2A^ was not different from the wt full-length receptor. Statistical analysis was performed using paired t-tests between I_max_ [-PS] and I_max_ [+PS] (*). Potentiation of the different mutants was analyzed by one-way ANOVA followed by Dunnett´s multiple comparisons test (^#^). **(C)** Dose-response analysis of PS for wt GluN1/GluN2A, GluN1^ΔM4^+M4^N1^/GluN2A, and GluN1^ΔM4^+M4^N1-M818A^/GluN2A showing nearly equal EC_50_ values of GluN1/GluN2A and GluN1^ΔM4^+M4^N1^/GluN2A (12.4 ± 1.3 µM, *n* = 5 and 15.0 ± 1.1 µM, n = 3; *p* < 0.05). The M4^N1-M818A^ mutation resulted in a significant decrease in PS affinity (EC_50_: 23.2 ± 1.4 µM; n = 4; *p* < 0.001) compared to the non-mutated M4^N1^. **(D)** Quantification of I_max_ potentiation of PS for GluN1/GluN2A, GluN1^M813A^/GluN2A and GluN1^M818A^/GluN2A showed significantly increased potentiation for both mutants compared to wt (M813A 1.40 ± 0.09-fold; *n* = 9; *p* < 0.05 and for M818A 1.72 ± 0.19-fold; *n* = 12; *p* < 0.01 compared to wt 1.10 ± 0.09-fold; *n* = 8). Comparison of GluN1^ΔM4^+M4^N1^/GluN2A and GluN1^ΔM4^+M4^N1^/GluN2D showed a significant difference in PS potentiation (2.89 ± 0.2-fold, *n* = 8 compared to 1.45 ± 0.19-fold, n = 11; *p* < 0.0001). The normally inhibitory overall effect of PS on GluN1/GluN2D was converted to a potentiating one for GluN1^ΔM4^+M4^N1^/GluN2D. Statistical analysis was performed using paired t-tests between I_max_ [-PS] and I_max_ [+PS] (*). Potentiation of the different mutants was analyzed by one-way ANOVA followed by Dunnett´s multiple comparisons test (^#^). **(E)** Representative TEVC uptake of the inhibition of GluN1/GluN3A by PS. Dose-response curves of PS at GluN1/GluN3A, GluN1^M813A^/GluN3A, GluN1^F817A^/GluN3A, GluN1^M818A^/GluN3A showing no differences between wt and the F817A mutant, but a strong decrease in PS affinity at the M813A and M818A mutants (111 ± 11 μM, *n* = 4; *p* < 0.0001 and 19.5 ± 1.7 µM, *n* = 4; *p* < 0.0001 compared to 8.5 ± 0.9 µM, n = 7 for wt). Data represent mean ± SEM.

To investigate the importance of our identified interactions of the GluN1 M4 fragment with the M1 and M3 of the neighboring subunit for the functional modulation of GluN1/GluN2A receptors, we performed I_max_ measurements in the absence and presence of the neurosteroid pregnenolone sulfate (20-oxo-5-pregnen-3β-yl sulfate, abbreviated PS; Horak et al., 2006; Jang et al., 2004; Krausova et al., 2020; Wilding et al., 2016) after expression of the wt-GluN1/GluN2A receptor and the GluN1^ΔM4^+M4^N1^/GluN2A and GluN1/GluN2A^ΔM4^+M4^N2A^ constructs at saturating agonist concentrations. Remarkably, when I_max_ values were compared in the absence and presence of PS for the different constructs, a difference in the modulation of whole-cell currents was immediately apparent. In contrast to the GluN1/GluN2A and GluN1/GluN2A^ΔM4^+M4^N2A^ receptors, where PS at a concentration of 100 µM potentiated maximal agonist-inducible whole-cell currents of 2.8 ± 0.7 µA (*n* = 8) and 0.059 ± 0.02 (*n* = 7) only maximally by ca. 1.1-fold, respectively, in GluN1^ΔM4^+M4^N1^/GluN2A receptors the I_max_ value of 0.146 ± 0.056 µA (*n* = 5) was extremely increased by 2.9 ± 0.2-fold in the presence of PS (t (14) = 8.74; *p* < 0.0001; Figures 4A,B). Analysis of M4^N1^ mutants M813A, F817A, M818A, and L819A in the presence of PS revealed an increase in the I_max_ of 0.036 ± 0.007 µA (*n* = 5) for M4^N1-F817A^ by 2.7 ± 0.12-fold, for M4^N1-M818A^ of 0.034 ± 0.004 µA (*n* = 6) by 5.9 ± 1.2-fold, and for M4^N1-L819A^ of 0.033 ± 0.012 µA (*n* = 5) by 2.6 ± 0.2-fold after co-expression with GluN1^ΔM4^/GluN2A (Figure 4B). No current could be measured for GluN1^ΔM4^+M4^N1-M813A^/GluN2A even after PS addition (*n* = 7, data not shown), again highlighting the particular importance of the M813 position for M4 binding to the GluN1^ΔM4^/GluN2A core receptor. To decipher possible differences in the mechanism of PS modulation of GluN1/GluN2A and GluN1^ΔM4^+M4^N1^/GluN2A-mediated currents, we analyzed glutamate dose-response curves in the presence of potentiating PS concentrations. This revealed that PS induced a similar 2-fold increase in apparent glutamate affinity for both GluN1/GluN2A and GluN1^ΔM4^+M4^N1^/GluN2A receptors (see Supplementary Figure 2 Suppl. data). Thus, a shift in glutamate EC_50_ value from 4.2 ± 0.47 µM to 1.87 ± 0.29 µM and from 2.3 ± 0.2 µM to 1.31 ± 0.2 µM was observed for GluN1/GluN2A and GluN1^ΔM4^+M4^N1^/GluN2A receptors, respectively. Similarly, analysis of PS potentiation affinity for the GluN1/GluN2A and GluN1^ΔM4^+M4^N1^/GluN2A receptors revealed nearly similar EC_50_ values of 12.4 ± 1.3 µM and 15 ± 1.1 µM, with a small but significant decrease in PS affinity for GluN1^ΔM4^+M4^N1^/GluN2A [Figure 4C; t (6) = 2.873; *p* = 0.0283]. Interestingly, the M4^N1^ mutant M818A, which showed the strongest increase in I_max_ of 5.93 ± 1.21-fold in the presence of PS (see Figure 4B), caused a significant decrease in PS affinity to 23.2 ± 1.4 µM [t (7) = 12; *p* < 0.0001]. Analysis of GluN1^M813A^/GluN2A and GluN1^M818A^/GluN2A full-length constructs also showed significantly increased I_max_ potentiation for both compared to wt (1.40 ± 0.09-fold and 1.72 ± 019-fold; Figure 4D). Since there is evidence for a balance of positive- and negative-modulatory (PAM and NAM) neurosteroid recognition sites leading to PS potentiation in GluN1/GluN2A and GluN1/GluN2B receptors and PS inhibition in GluN1/GluN2C and GluN1/GluN2D receptors (Horak et al., 2006), we examined the effect of PS on the modulation of GluN1/GluN2D and GluN1^ΔM4^+M4^N1^/GluN2D receptors. Significantly, the inhibitory effect of PS at GluN1/GluN2D was converted to a potentiating one at GluN1^ΔM4^+M4^N1^/GluN2D receptors by increasing an I_max_ value of 0.025 ± 0.003 µA (*n* = 11) by 1.4-fold (Figure 4D). These data imply two possibilities; That either 1) the NAM effect of PS in GluN1^ΔM4^+M4^N1^/GluN2 receptors is attenuated by a change in the interactions of the M4 of GluN1 with the TMs of neighboring GluN2, and consequently there is enhanced potentiation by PS at the PAM-binding site, or 2) the PAM effect of PS in GluN1^ΔM4^+M4^N1^/GluN2 receptors is enhanced by a change in the interactions of the M4 of GluN1 with the TMs of the neighboring GluN2, resulting in potentiation.

To obtain evidence for a possible NAM or PAM-binding site for PS in the M4 region of GluN1, we examined the effect of PS at glycine-gated GluN1/GluN3 receptors. Thus, in the absence and presence of PS, we performed I_max_ measurements after expression of the wt GluN1/GluN3A receptor following application of the agonist glycine (10 mM) in combination with the potentiating ligand MDL-29951 (0.2 µM). Remarkably, when comparing I_max_ values in the absence and presence of 100 μM PS for the different constructs, a difference in the modulation of whole cell currents of GluN1/GluN2A and GluN1/GluN3A receptors was immediately apparent. In contrast to GluN1/GluN2A receptors, where PS at a concentration of 100 µM potentiated maximal agonist-inducible whole-cell currents (Figure 4A), I_max_ values of GluN1/GluN3A receptors were extremely decreased in the presence of PS (Figure 4E). Affinity analysis of PS inhibition for GluN1/GluN3A receptors revealed an IC_50_ value of 8.5 ± 0.9 µM (*n* = 7), remarkably a similar value to the EC_50_ value of 12.4 ± 1.3 µM for GluN1/GluN2A receptors. Interestingly, GluN1^M813A^ and GluN1^M818A^ mutants showed a strong increase in the IC_50_ value of PS to 111 ± 11 μM and 19.5 ± 1.7 µM, respectively [t (9) = 25.43; *p* < 0.0001 and t (13) = 15.22; *p* < 0.0001; Figure 4E]. In contrast, expression of GluN1^F817A^/GluN3A receptors resulted in an unchanged IC_50_ value for PS of 6.73 ± 1.73 µM [t (8) = 2.178; *p* = 0.061]. Thus, our analysis of GluN1^M813A^/GluN3A and GluN1^M818A^/GluN3A receptors revealed strong evidence for a NAM binding site for PS at the interface of the M4 of the GluN1 subunit with the adjacent GluN3A subunit. Considering the assumption of a PAM and NAM neurosteroid recognition site in GluN1/GluN2 receptors, the increase in PS potentiation of our GluN1-M4 mutations in GluN1/GluN2A receptors implies an impairment of inhibition by the NAM-PS binding-site at the interface of the M4 of the GluN1 subunit to the neighboring GluN2A subunit. Consequently, impairment of inhibition by PS leads to an increase in potentiation by PS at the corresponding PAM-binding site. Thus, our results suggest a specific role of amino acid residues in GluN1-M4 for its binding to the core receptor, which are involved in the negative modulatory effect of the neurosteroid PS.

## Discussion

The importance of the evolutionary new M4 transmembrane segment in ionotropic glutamate receptor function and assembly is still not clearly understood. In the present study on the role of M4 in NMDAR functionality, we show, after heterologous expression in *Xenopus* oocytes, that deletion of M4 in the GluN1, GluN2A, and GluN3A subunits results in nonfunctional membrane receptors despite preserved receptor assembly and surface expression. Remarkably, co-expression of the corresponding M4 segments of GluN1 and GluN2A, but not the GluN3A subunit, as an isolated peptide in M4-deleted GluN1/GluN2A receptors rescued receptor function without altering the apparent agonist affinities of glutamate and glycine. The substitution of non-conserved residues within the putative interfaces of M4 of GluN1 with neighboring GluN subunits suggests a specific role for these residues in 1) the functional coupling of isolated-expressed M4 fragments to the core receptor and 2) the negative modulatory effect of the neurosteroid pregnenolone sulfate, underscoring the importance of M4 and its interactions in the regulation of NMDA receptor function.

### The Role of M4 in NMDA Receptor Assembly and Function

In iGluRs, the M4 of one subunit is structurally linked to the pore-forming M1 and M3 helices of the neighboring subunit (Sobolevsky et al., 2009; Karakas and Furukawa, 2014). Based on this exclusive interaction of the peripheral M4 with the central M1 and M3 segments of the neighboring subunit, an interaction of residues of the M4 with these transmembrane segments has been proposed to mediate receptor assembly or at least surface targeting of iGluRs (see for example Amin et al., 2017). Indeed, some results showed that the peripheral M4 helix is involved in subunit association or at least confers additional stability to the tetrameric receptor, ideas based mainly on AMPAR assembly studies (Amin et al., 2017; Gan et al., 2016; Salussolia et al., 2011, 2013). In contrast, studies on conventional GluN1/GluN2 NMDA receptors led to the view that the M4 segment is more required for the formation of GluN1/GluN2 heterodimers and, in the case of the GluN2B subunit, also for masking ER retention signals in GluN1 (Horak et al., 2008; Cao et al., 2011). We can show that neither single nor double M4 deletions affect the assembly and surface expression of the conventional GluN1/GluN2A NMDAR after heterologous expression in oocytes, supporting the view that the M4 of glutamate-gated NMDARs are structural determinants more likely to be involved in the allosteric regulation of ion channel opening by modulatory compounds (Krausova et al., 2020). The absence of M4 in prokaryotic GluR0 also supports the idea that this transmembrane region may not be essential for NMDAR assembly and that its presence in eukaryotic iGluRs is predominantly required to modulate or fine-tune the kinetic properties of the channel. Consistent with this conclusion, our current study also shows for the less studied glycine-gated GluN1/GluN3A NMDAR that deletion of M4 does not affect assembly or cell surface expression. In summary, our data support the view that the M4 segment, at least after heterologous expression in oocytes, is not required for oligomerization of glutamate- or glycine-gated NMDARs.

Thus, although the M4 is not involved in NMDAR assembly and surface trafficking, it is essential for receptor function. This is impressively demonstrated by the rescue of channel function of M4-deleted GluN1/GluN2A receptors with unaltered apparent agonist affinity after co-expression with the corresponding M4 fragment as an isolated peptide. Surprisingly, co-expression of M4 in GluN1/GluN3A M4-deleted constructs resulted in a complete loss of receptor subunit expression, although all M4 fragments tested were expressed and incorporated into the cell surface with equal efficiency. We interpret this pronounced instability of M4-deleted GluN1/GluN3A receptor proteins in the presence of the corresponding M4 as indicating rapid degradation of these receptors in the ER by quality control mechanisms. In contrast, for the M4-deleted GluN1/GluN2A receptors, we found retained surface expression after co-expression with the corresponding M4, which, however, is accompanied by a decrease in maximal inducible whole cell currents, a finding already described previously (Schorge and Colquhoun, 2003). This may be due to a reduced likelihood of selective interaction of expressed core receptors and isolated M4 fragments. This is also supported by our finding that co-expression of the M4 of GluN1 could also rescue GluN1/GluN2A^ΔM4^ receptors, whereas the less conserved M4 segment of the GluN3A subunit in GluN1^ΔM4^/GluN2A or GluN1/GluN2A^ΔM4^ could not restore receptor function. This ability of M4 fragments to differentially rescue the functionality of M4-deleted GluN1/GluN2 receptors could be determined by the individual exchange of amino acid residues in the interface of M4 with neighboring TMs. Thus, exchange of the residue methionine 813 conserved in the GluN1 and GluN2 subunits, which is associated with refractory seizures and global developmental delay when exchanged to valine in the GluN2A subunit (Chen et al., 2017; Venkateswaran et al., 2014), results in decreased I_max_ when co-expressed with M4-deleted receptors, again suggesting a specific role of interactions in functional coupling of M4 to the core receptor. This is also supported by our finding that the nearby residue F637 in M3 of the neighboring GluN2 subunit also decreased the I_max_ of M4-deleted receptors. Consistent with our findings, the insertion of large residues in a 2017 study by Amin and colleagues in a tryptophan scan of the M4 interface of the GluN1 and GluN2A subunits also confirms decreased functionality of the M4 interfaces.

Overall, we identified amino acid residues important for the restoration of receptor function by isolated M4 peptides. The formation of a disulfide bond after cysteine substitution (GluN1-M813C and GluN2A-F637C) suggests a specific interaction of our GluN1 M4 fragment with the M3 of the GluN2A subunit as an interaction partner. A second interaction could be between GluN1-F817 of M4 and position M560 at M1 of the GluN2A subunit because cysteine substitution at the two residues (M4^N1-F817C^ and GluN2A^M560C^) likewise allowed insertion of a disulfide bond. This assumption is supported by the fact that GluN1^ΔM4^+M4^N1-F817A^/GluN2A shows a strong reduction of the rescue effect (∼90%), although not a complete loss of function as in M4^N1-M813A^. Moreover, GluN1-F817 is known to be disease-associated with phenylalanine-to-leucine exchange, leading to intellectual and mental disability, highlighting its importance for NMDAR functionality (Lemke et al., 2016). Steric restriction of the TM interface by oxidative cross-linking in the GluN1^ΔM4^+M4^N1-M813C^/GluN2A^F637C^ and GluN1^ΔM4^+M4^N1-F817C^/GluN2A^M560C^ receptors resulted in loss of function in both, suggesting that the receptor interface requires some degree of flexibility. Our assumptions are consistent with 1) structural analyses of GluN1/GluN2 receptor complexes showing a unique arrangement of M4 with distinct intersubunit interactions (Lee et al., 2014; Lü et al., 2017) and 2) Wollmuth lab molecular dynamics simulations showing that the tip of the M4 helix must move to stabilize the NMDAR open state (Amin et al., 2018). Based on the results presented here, we propose that the main role of M4 in NMDARs is to ensure the functionality of agonist-induced channel opening. Our results clearly show that M4 is not involved in the efficiency of assembly; rather, M4 with its interactions represents an important segment for conformational changes within TMs for channel opening and its modulation after ligand binding at the interface of TMs.

### The Role of M4-TM Interfaces in Determining the Efficiency of Pregnenolone Sulfate Modulation on NMDA Receptor

The importance of interactions at TM interfaces in binding modulators and modulating receptor response in NMDARs is poorly understood. As mentioned above, M4 interfaces are thought to alter the kinetic properties of conventional NMDARs by rearranging the M1 and M4 helices, thereby stabilizing the open-state position of the M3 helices (Amin et al., 2017, 2018). In addition, NMDAR channel function is likely to be strongly modulated by the repositioning of peripheral M4 segments through interactions with lipids or through binding sites for positive and negative modulators (Casado and Ascher, 1998; Traynelis et al., 2010; Ren et al., 2012; Korinek et al., 2015; Wilding et al., 2016). We can show that the M813A interface mutation in M4 of GluN1 specifically decreases inhibition of GluN1/GluN2A, and particularly pronounced, at GluN1/GluN3A receptors by PS. Our results further clearly demonstrate that the M4s of GluN1 and GluN2 subunits may not be equally involved in determining neurosteroid efficiency; rather, the M4 of GluN1 represents an important segment for the negative effect of PS. Consequently, we attribute the complete lack of a positive-modulatory effect of PS in GluN1/GluN3A receptors to the unique design of the M4 interface of the GluN3 subunit. Our results are consistent with other studies on conventional GluN1/GluN2 NMDA receptors, in which it has been shown that the M4 and its linker region of GluN2 subunits in particular control the subunit-specific PS action by determining the positive modulatory effect of PS (Jang et al., 2004; Krausova et al., 2020). However, the exact mechanism that couples the modulatory properties of M4 to channel activation and the importance of these TM interfaces in subunit-specific PS regulation are still unknown. In the absence of detailed information on the structure and conformation of the M4 interface upon binding of a modulator within TMs, we hypothesize that specific side-chain interactions within the M4/TM regions are important for positive- or negative-regulatory interactions. We think that our proposed negative-modulatory steroid interaction site, formed by the M4-GluN1 and M1/M3-GluN2 or GluN3 helices, undergoes a structural rearrangement after PS binding and thus negatively-allosterically affects channel conformation. Ultimately, this negative-modulatory effect would reduce the potentiating effect of a second, separate steroid-binding site. Based on the results presented here, we therefore propose that 1) the flexibility and positioning of the M4 of GluN1 is important for the inhibitory effect of PS, and 2) that the ratio of the effects of the positive- and negative-modulatory steroid-binding sites determines the subunit-dependent modulation of GluN1/GluN2/3 receptors by PS.

## Conclusion

The present study demonstrates a prominent role of the M4 of GluN1 in determining PS efficacy at NMDARs, because mutations within the TM interfaces result in a strong loss of PS-induced inhibition in GluN1/GluN3A receptors. Taken together, our results implicate distinct roles of M4 segments in different NMDAR subunits and highlight their importance in the regulation of NMDAR function by neurosteroids. Compounds with PS-like properties targeting the GluN1-M4 interfaces may represent powerful tools for selective modulation of glutamate- and glycine-activated NMDA receptors *in vivo*.

## Data Availability

The raw data supporting the conclusion of this article will be made available by the authors, without undue reservation.
