# Supplementary material for: Analysis of M4 Transmembrane Segments in NMDA Receptor Function: A Negative Allosteric Modulatory Site at the GluN1 M4 is Determining the Efficiency of Neurosteroid Modulation
Source: Front Pharmacol. 2021 Oct 1;12:769046. doi: 10.3389/fphar.2021.769046 (PMC8517087; doi:10.3389/fphar.2021.769046)
Supplement: Supplementary file 1 [file DataSheet1.PDF]

Supplement

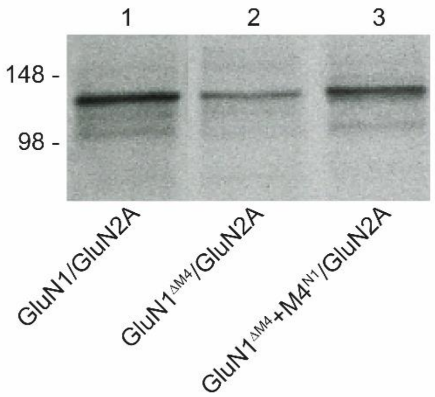

Fig. 1 Impact of M4-truncation and M4-segement coexpression on GluN1/GluN2A receptor expression.

SDS-PAGE of metabolic [35S]methionine-tagged GluN1/GluN2A, GluN1$^{\Delta M4}$/GluN2A and GluN1$^{\Delta M4}$+M4^{N1}/GluN2A receptors with purification of C-terminal His-tagged receptor subunit constructs by metal affinity chromatography. All constructs were properly expressed, M4-Segement coexpression did not alter GluN1$^{\Delta M4}$+M4^{N1}/GluN2A expression level.

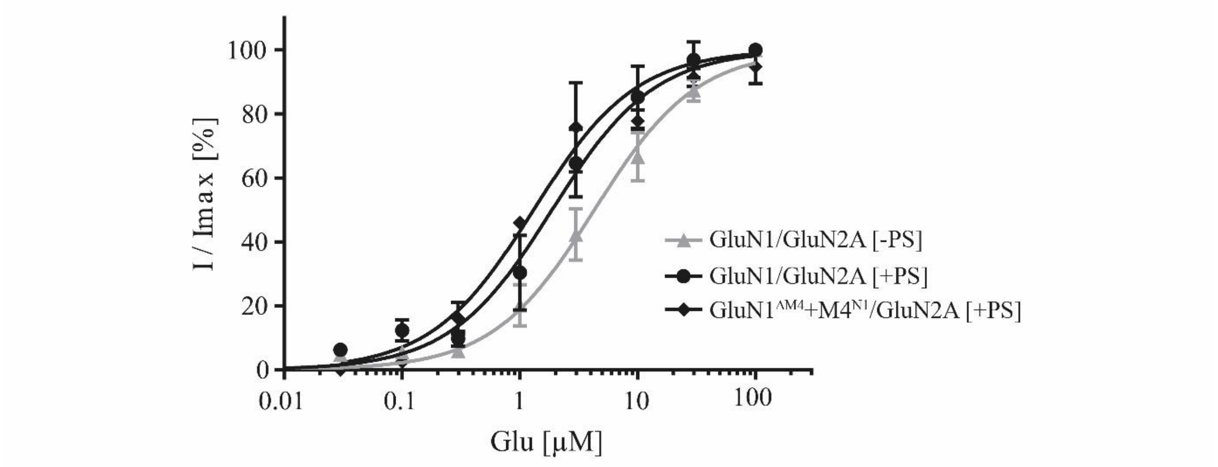

Fig.2 Impact of PS on the Glutamate Affinity.

Dose-response analysis showed that agonist affinity of GluN1/GluN2A and $GluN1^{\Delta M4}$+$M4^{N1}$/GluN2A were similar without PS modulation (see results Fig. 1C). PS modulation of both GluN1/GluN2A and $GluN1^{\Delta M4}$+$M4^{N1}$/GluN2A resulted in an increase of agonist affinity (GluN1/GluN2A [-PS] EC_{50}: 4.2±0.47 µM to [+PS] EC_{50}: 1.87±0.29 µM ($t(5) = 7.731$; $p= 0.0006$; and $GluN1^{\Delta M4}$+$M4^{N1}$/GluN2A [+PS] EC_{50}: 1.31±0.2 µM; $t(6) =11.64$; $p <0.0001$). The results show a similar shift of the agonist affinity for both wt and M4-segment coexpression. Statistics done by unpaired t-test. Data represent mean ±SEM.
